# How is patient involvement measured in patient centeredness scales for health professionals? A systematic review of their measurement properties and content

**DOI:** 10.1186/s12913-018-3798-y

**Published:** 2019-01-08

**Authors:** Eline Ree, Siri Wiig, Tanja Manser, Marianne Storm

**Affiliations:** 1 SHARE – Centre for Resilience in Healthcare, Faculty of Health Sciences, University of Stavanger, Stavanger, Norway; 2 FHNW School of Applied Psychology, University of Applied Sciences and Arts Northwestern Switzerland, Olten, Switzerland

**Keywords:** Patient centeredness, Patient involvement, Quality improvement, Systematic review, Measurement

## Abstract

**Background:**

Patient centeredness is an important component of patient care and healthcare quality. Several scales exist to measure patient centeredness, and previous literature provides a critical appraisal of their measurement properties. However, limited knowledge exists regarding the content of the various scales in terms of what type of patient centeredness they represent and how they can be used for quality improvement. The aim of this study was to explore the measurement properties of patient centeredness scales and their content with a special focus on patient involvement, and assess whether and how they can be used for quality improvement.

**Methods:**

A systematic review of patient centeredness scales was conducted in Medline, CINAHL, Embase, and SCOPUS in April and May 2017. Inclusion criteria were limited to articles written in English published from 2005 to 2017. Eligible studies were critically appraised in terms of internal consistency and reliability, as well as their content, structural, and cross-cultural validity. Type of studies included were scale-development articles and validation studies of relevant scales, with healthcare personnel as respondents. We used directed content analysis to categorize the scales and items according to Tritter’s conceptual framework for patient and public involvement.

**Results:**

Eleven scales reported in 22 articles were included. Most scales represented individual, indirect, and reactive patient involvement. Most scales included items that did not reflect patient centeredness directly, but rather organizational preconditions for patient centered practices. None of the scales included items explicitly reflecting the use of patient experiences of quality improvement.

**Conclusions:**

There is a lack of patient centeredness scales focusing on direct and proactive involvement of patients in quality improvement. It would be useful to develop such instruments to further study the role of patient involvement in quality improvement in healthcare. Furthermore, they could be used as important tools in quality improvement interventions.

**Electronic supplementary material:**

The online version of this article (10.1186/s12913-018-3798-y) contains supplementary material, which is available to authorized users.

## Background

The Institute of Medicine regards patient centered care and patient experiences as one of six dimensions of healthcare quality [1], and it is extensively addressed in the research literature [2]. The concept of patient centered care has received widespread attention in the scientific arena since the mid-1950s [3]. However, it became embedded in healthcare policies and regulations in several developed countries only at the turn of the millennium [4–7]. Although the focus on patient centered care has increased, the rationale, measurement, and implementation of strategies to improve patient centered care or to use patient experiences for quality improvement purposes have been widely debated [3, 8].

The Institute of Medicine defines patient centeredness as *“providing care that is respectful of and responsive to individual patient preferences, needs, and values and ensuring that patient values guide all clinical decisions”* [1]. However, the growing number of studies on this concept are inconsistent in terms of definitions, labels, understandings, and measures of patient centeredness [9]. Patient centeredness is, depending on the context, often used interchangeably with terms such as client-centered, user-centered, or person-centered [10]. Patient involvement is often mentioned as a condition for patient centeredness and seen as a strategy to achieve a patient-centered care [10]. Others consider patient involvement to be a dimension of patient centeredness [11]. In any case, patient involvement is higly related with patient centeredness and the concepts should be seen as interrelated rather than as independent from each other [10]. We consider patient involvement an important dimension of patient centeredness, and scales addressing the latter should also include some aspects of patient involvement.

Several measurement scales exist to measure patient centeredness from patients’ perspective [12]. Previous reviews of patient centered measurement scales have focused mainly on the measurement properties of instruments with little or no qualitative content analysis of the scales [12–15]. Two narrative reviews of the measures of person-centered care [14, 15] also lack a critical scrutiny of the research underpinning the included measurements. The purpose of Wilberforce et al.’s [13] systematic review was to identify, describe, and critically appraise measures of person-centeredness relevant to the long-term care of older people. The current review focuses on the measures of patient-centeredness from the staff members’ perspective, not limited to older adults, but validated in healthcare settings. Identifying and evaluating questionnaire-based scales from the staffs’ perspective has important practical implications. Health professionals are a key stakeholder in quality improvement work and often the target of quality improvement interventions utilizing pre- and post-intervention surveys. Furthermore, the field of quality and safety in healthcare is continuously expanding; thus, new measures were available since the previous reviews were published. Most importantly, none of the previous reviews have conducted a content analysis to explore the content of the scales in terms of what type of involvement they represent. To our knowledge, no previous studies have explored the content of patient centeredness scales in terms of whether and how patient involvement is represented.

The current review will be based on Tritter’s [16] definition of and conceptual framework for patient and public involvement (PPI), focusing on the patient involvement part. Tritter [16] has proposed a framework of ‘patient and public involvement (PPI)’ which he defines as *“the ways in which patients can draw on their experience and members of the public can apply their priorities to the evaluation, development, organization and delivery of health services.”* This definition emphasizes the potential of considering patients’ experiences in quality improvement work, which is specified by Tritter [16] as involvement that has an impact on healthcare practice, professional cultures, or organizational policy. Tritter [16] suggested three dimensions of involvement: a) direct or indirect, b) individual or collective, and c) reactive or proactive. Direct involvement refers to patients or the public taking part in actual decision-making, such as deciding what treatment to use (individual direct involvement) or being part of a support group in designing a new treatment (collective direct involvement). Indirect involvement implies gathering information through patient experience surveys or reports from support groups, for example; however, it is up to the health professionals to decide whether or how to act upon this information. Reactive involvement implies that patients or the public are responding to a pre-existing agenda while proactive involvement implies that they contribute to shaping the agenda.

Based on previous reviews and our understanding of patient involvement as an important dimension of patient-centredness, we argue that there is a lack of scales that measure solely patient involvement and do so from the perspective of healthcare personnel [17]. We originally planned to review patient involvement scales with health professionals as respondents, but a preliminary search in scientific databases revealed a lack of studies reporting on this. Therefore, we decided to review patient centeredness scales. The aims of this study were to 1) conduct a quality appraisal focusing on the measurement properties of patient centeredness scales for healthcare professionals, 2) explore the content of the scales in terms of whether and how patient involvement is represented according to the PPI conceptual framework, and 3) explore whether the scales reflect patient involvement in quality improvement practices. More specifically, we explored the degree to which scales and specific items designed to measure patient centeredness included aspects of individual involvement or collective involvement as well as whether the involvement was direct or indirect and reactive or proactive, according to Tritter’s [16] conceptual framework. We also explored whether the scales reflected patient involvement in quality improvement practices.

## Methods

### Design

We conducted a systematic review using the Preferred Reporting Items for Systematic reviews and Meta-Analyses (PRISMA) check-list [18].

### Search strategy

We searched the following databases: Medline, CINAHL, Embase, and Scopus. A combination of keywords, mesh-terms, and subject headings was used in all searches. The searches in the databases were structured around three main concepts: psychometrics, patient-centeredness and involvement, and quality improvement. Regarding patient centeredness, in addition to patient-, we used the pre-fixes person- and client- along with the suffixes –involvement, centeredness, centered care, oriented, participation, and experiences, in addition to individual* care (UK and US spelling) (see Additional file 1 for description of the systematic search strategy). Terms related to psychometrics, such as validity and reliability, were used to ensure that the articles reported on measurement scales. To identify articles relevant for quality and safety in healthcare, we used the following terms: quality improvement, quality of healthcare, and patient safety. Each search was adapted to the specific database used according to mesh-terms. All authors met to discuss and revise the search strategy several times before deciding on the final search. A research librarian assisted with the technical parts of the search. Because the concept of patient centeredness and patient involvement in healthcare has developed considerably in the last decade [17], with increasing focus on the patient as a key actor in healthcare, and to manage the volume of articles identified, the search was limited to articles in English published as of 2005. Furthermore, we screened the reference lists of the included articles and previous reviews to reveal if there were relevant scales published before 2005 that needed to be included.

### Study selection and eligibility criteria

**Fig. 1:**
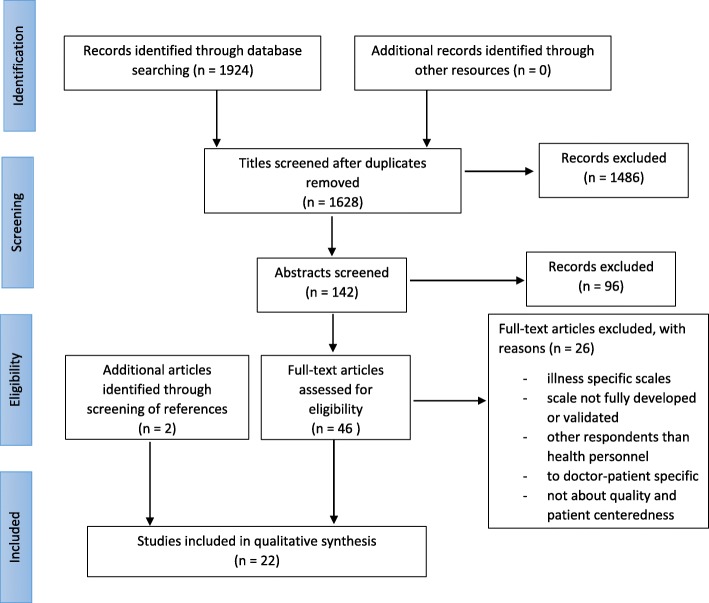
PRISMA flowchart of article selection

The first electronic search was undertaken in April 2017 and updated in May 2017. Once duplicates were removed, two researchers (ER and MS) assessed titles and abstracts of the identified articles independently, and they retrieved the articles that were considered eligible for full screening (see Fig. 1). Uncertainty emerged concerning the inclusion of six of the articles; thus, a third researcher (SW) was involved in discussions of these until consensus was reached.

Three eligibility criteria guided the selection of articles. First, selected articles had to report on the development and/or validation of questionnaire-based measurement scales. Second, the scales had to represent the perspective of health personnel. Third, the scales had to measure patient centeredness or involvement in healthcare settings. However, disease specific scales, highly consultation specific tools that doctors use to map his/her patients’ needs or preferences, scales that were not fully developed or validated, and studies that included respondents other than health personnel or those that did not focus on patient centeredness were excluded from the analysis. We also excluded administrative checklist tools that are used in practice, for example to map the status quo regarding patient centeredness practices in an organization, and are not suitable to be used as measurement scales in research [19].

### Data extraction and critical appraisal

For the critical appraisal of the methodological quality of the 22 included studies, we evaluated the quality of measurement properties using five of the seven criteria applied by Wilberforce et al. [13]. Specifically, we assessed the instruments’ internal consistency, its reliability, as well as its content, structural, and cross-cultural validity. We considered these as clear criteria for assessing measurement properties against established thresholds of acceptability, and using the same approach as Wilberforce et al. [13] will also allow comparison of results. We did not include the two measurement properties “measurement error” and “hypothesis testing”. We argue that these are not standard quality indicators for measurement scales. For example, measurement error is usually very difficult to calculate, and we would not gain much information for the review from this rating. Many of the included articles are scale development articles, in which hypothesis testing is not relevant.

We compared each instruments’ measurement properties against established acceptability thresholds. Based on our initial coding experience, we decided to score internal consistency for the overall scale and its sub-scales separately. All studies were scored as either meeting (+) or not meeting (−) the threshold for acceptability (Table 1) or as not reporting results on a criterion (NR). To ensure a fair comparison between studies, we added “not applicable” (NA) as a coding option, since some studies did not meet all criteria (e.g., cross-cultural validity in initial scale development studies and content validity of translated versions of the original scale). Two members of the research team (MS, TM) who independently reviewed all studies conducted the critical appraisal. All discrepancies were then discussed with a third research team member (ER) after which a consensus rating was obtained. Furthermore, we extracted data on subscales, study setting and the articles’ use of a conceptual framework (Table 2). Regarding the latter, we were open as to which specific framework was used. However, we deemed it important that the study did use a conceptual framework, definitions or operationalizations that were used when developing the scales.

**Table 1:** Quality criteria and thresholds of acceptability for measurement properties (adapted from Wilberforce et al., 2016)

Measurement property	Rating	Threshold for acceptability
Internal consistency: overall scale	+	Cronbach alpha >.70 and < .95
	–	Cronbach alpha <.70 and > .95
Internal consistency: sub-scales	+	Cronbach alpha >.70 and < .95
	–	Cronbach alpha <.70 and > .95
Reliability	+	Intraclass Correlation Coefficient (ICC) > .70 OR Pearson’s Correlation Coefficient (r) > .80
	–	ICC < .70 OR *r* < .80
Content validity	+	Assessed in target population that items are a complete representation of concept under measurement and that all items are relevant
	–	Questionnaire is incomplete OR contains irrelevant items
Structural validity	+	Factors explain 50% of variance
	–	Factors explain less than 50% of variance
Cross-cultural validity	+	Original factor structure confirmed OR no differential item functioning
	–	Original factor structure not confirmed OR important differential item functioning observed

### Content analysis

The instruments and items were categorized according to Tritter’s [16] conceptual framework (individual vs collective involvement, and reactive vs. proactive involvement) using directed content analysis [20]. More specifically, ER and MS reviewed individual items from each instrument and determined to which of Tritter’s categories they belonged. Furthermore, ER and MS assessed instruments according to whether they reflected patient involvement in quality improvement practices, i.e., whether patients are actually involved in developing or changing service practices related to quality. SW was included in the final round of discussions based on which agreement about categorization was reached.

## Results

### Study selection

Out of 1628 titles, 142 were retained for abstract screening (see Fig. 1) and 46 of these were retained for full-text screening. Twenty articles from the systematic search were retained in the review. In addition, two articles were found and retained through screening of the reference lists in two of the included articles. The reference lists of previous literature reviews on patient centeredness scales were also screened, but no relevant articles that had not already been captured in our search were found.

### Descriptive information of the included scales

The review identified 11 instruments reported in 22 articles. The descriptive information of the included scales and articles is presented in Table 2, including scale information, study populations, settings, and use of a conceptual framework. Scale abbreviations are also explained in Table 2.

**Table 2:** Overview of included instruments

Name of measure	No. of items	Subscales	Study settings	Conceptual framework^a^
Person-Centred Climate Questionnaire – Staff version (PCQ-S) [22, 36, 37, 50]	14	1. A climate of safety 2. A climate of everydayness 3. A climate of community	Hospital wards at three hospitals in northern Sweden [22] Australian hospital facility [36] Nursing homes in the eastern part of Norway [37] Traditional nursing homes and special care units for people with dementia, Sweden [50]	Not included. Referred to several definitions and conceptualizations in the literature without making explicit which one they used.
Patient-Centred Care Competency (PCCC) [39]	17	1. Respecting patients perspectives 2. Promoting patient involvement in care processes 3. Providing for patient comfort 4. Advocating for patients	Two teaching hospitals in Seoul, Korea	Sees patient-centered care as a global concept that includes knowing and respecting patients’ values, preferences and needs; providing for patients’ physical and emotional comfort; advocating for patients; and promoting partnership between providers and patients in care decisions.
Person-Centred Health Care for Older Adults Survey (PCHCOA) [25]	31	Eight dimensions, not labeled	17 health services (community rehabilitation centers, continence clinics, general medical, geriatric evaluation and management unit, other) across Victoria, Australia	Identified five principles underlying person-centered health care based on which the scale was developed: getting to know the patient, sharing power and responsibility, accessibility and flexibility, coordination and integration, and having an environment that is conductive to person-centered care
Person-Centered Care Assessment Tool, (P-CAT) [24, 33, 34, 51, 52]	13 p	1. Extent of personalizing care mount of organizational support 2. Degree of environmental accessibility	Long-term aged care facilities in Australia [24] Norwegian residential units for older people [33] Residential elderly care homes in Spain [34] Residental care units for older people in Sweden [51] Residential care facilities in north-western China [52]	Not included. Referred to several definitions and conceptualizations in the literature without making explicit which one they used.
Individualized care Scale (ICS-Nurse) [21, 29]	34	1. ICS-A-Nurse (Explores nurses’ view on how they support patient individuality through nursing activities in general) 2. ICS-B-Nurse (Explores the extent to which they perceive the care they provide to patients as individual). Both dimensions have three subscales: Clinical situation, personal life situation, and decisional control over care	Inpatient wards in one university, two regional and two psychiatric hospitals, and four health centers [21] One university, two central and two county hospitals [29]	Two dimensions of individualized care, as seen from nurses’ perspective, were used to develop the scale: 1) “by exploring nurses’ views about how they support their patient’s individuality through specific nursing activities”, and 2) “by examining how nurses evaluate the maintenance of individuality in the care they provide.”
Person-directed care (PDC) [28, 31, 38]	50	1. Knowing the person 2. Comfort care 3. Autonomy 4. Personhood 5. Support relations 6. Staff work with residents 7. Personal environment for residents 8. Management/structure	Long-term care settings (residential care, assisted living and home care settings) in Oregon [31] The Veterans Health Administration Community Living Center [28] Korean nursing homes [38]	Based on literature review and consultations with PDC experts (clinicians and providers), the authors identified six central dimensions of PDC: personhood, knowing the person, autonomy and choice, comfort care, nurturing relationship, and supportive environment. The scale development article provides thorough definitions of each dimension [31].
Self-efficacy in patient-centeredness (SEPCQ-27) [32]	27	1. Exploring the patient perspective 2. Sharing information and power 3. Dealing with communicative challenges	Two medical schools (Aarhus University, University of Southern Denmark) and two hospitals (Aarhus and Copenhagen University hospitals)	*Patient-centeredness* was defined by three core attributes: 1) consider the patients’ individual experiences, needs, and perspectives; 2) provide patients opportunities to participate in their care; 3) improve the relationship between patient and clinician.
Person-Centered Practices in Assisted Living (PC-PAL) – staff version [26]	62	1. Workforce practices 2. Social connectedness 3. Individualized care and services 4. Atmosphere 5. Caregiver-resident relationships	Assisted Residences in North Carolina (small, medium and large communities)	Used a conceptual framework set forth by the Center for Excellence in Assisted Living (CEAL) in their Informational Guide for Person-Centered Care in AL (that person-centeredness is built on core values and philosophy, relationships and community, management/ownership/governance, leadership, workforce, services, meaningful life, environment, and accountability).
Individualized Care Inventory (ICI) [27, 30]	Short version: 22 Full version: 46	1. Knowing the person/resident 2. Resident autonomy and choice 3. Communication (staff-to-resident communication, and staff-to-staff communication)	Long-term care facilities in Victoria and Sidney, British Columbia (BC), Canada [30] LTC facilities in British Columbia health authorities [27]	Literature review derived the following definition of individualized care that guided development the scale: Care that reflects 1) the individuality of the resident, i.e., knowing the person/resident, 2) an opportunity for autonomy and choice for the resident, and 3) open communication between staff themselves and between staff and residents.
Patient-centered care (PCC) [23]	27	1. Holistic care 2. Collaborative care 3. Responsive care	Acute care institutions in Ontario, Canada	Conceptualized PCC as holistic-, collaborative-, and responsive care based on an integrative review of conceptual, empirical, and clinical literature.
Geriatric Care Environment Scale (GCES) [35]	28	1. Aging-Sensitive Care Delivery 2. Resource Availability 3. Institutional Values Regarding Older Adults and Staff 4. Capacity for Collaboration	71 hospitals that are a part of a national program aimed at system improvement to achieve patient-centered care for older adults (Nurses Improving Care for Health System Elders (NICHE)) in New York.	Not included

^a^All information in this column is based on the original scale development study

Although all included instruments measured patient centeredness, the articles differed in conceptualization and use of conceptual frameworks. Some defined patient centeredness as a holistic concept and measured different kinds of patient centered care (ICS-N) [21] while others focused on organizational preconditions for patient-centered care (PCQ-S) [22] or a combination of patient centered care and preconditions (e.g., PCC [23], P-CAT [24], PCHCOA [25], PC-PAL [26]. Few articles explicitly used a conceptual framework to support the development of the instruments. Most of them conducted a review of the literature to develop a definition and identify the elements that should be included in the instrument development.

### Quality appraisal

The results of the quality appraisal of the 22 studies are presented in Table 3. The appraisal procedure revealed variation in the reporting of the key measurement properties across the studies. Internal consistencies of either subscales, total scale, or both were reported in twenty studies (91%), with the exception for the PCC [23] for which internal consistencies of subscales or total scales were not reported. Fifteen studies (68%) received high quality (+ rating) for internal consistency for total scale while six studies (27%) did not report on this measure [23, 27–31]. Reliability received high quality (+ rating) in six studies [21, 26, 30, 32–34], and low quality (− rating) in five studies (23%). Ten studies (45%) did not report reliability. Eleven scale development studies (50%) measured content validity, with eight studies (36%) receiving high quality (+ rating) [21–25, 30, 31, 35]. Structural validity was reported in 19 studies (86%), with fifteen studies (15%) reporting high quality (+ rating) with factors explaining 50% of variance. Cross-cultural validity was not applicable in eleven scale development studies, and only received high quality (+ rating) in four studies (18%) [29, 36–38].

**Table 3:** Quality appraisal

Scale	Study	Internal consistency		Reliability	Content validity	Structural validity	Cross-cultural validity
		subscales	total scale				
PCHCOA	Dow et al., 2013	–	+	NR	+	+	NA
PCCC	Hwang, 2015	+	+	–	–	+	NA
GCES	Kim et al., 2007	+	+	NR	+	+	NR
SEPCQ	Zachariae et al., 2015	+	+	+	–	+	NA
PC-PAL (staff version)	Zimmerman et al., 2015	+	+	+	–	+	NA
PCC	Sidani et al., 2014	NR	NR	NR	+	–	NA
ICI original	Chappell et al., 2007	–	NR	+	+	–	NA
ICI	O’Rourke et al., 2009	+	NR	NR	NA	+	NR
PCQ-S Swedish original	Edvardsson et al., 2009	+	+	–	+	+	NA
PCQ-S English	Edvardsson et al., 2010	–	+	–	NA	+	+
PCQ-Norwegian	Bergland et al., 2012	+	+	–	NA	+	+
PCQ-S Swedish	Edvardsson et al., 2015	+	+	NR	NA	NR	NR
P-CAT English original	Edvardsson et al., 2010	–	+	–	+	+	NA
P-CAT Norwegian	Rokstad et al., 2012	+	+	+	NA	–	NR
P-CAT Swedish	Sjögren et al., 2012	+	+	–	NA	–	NR
P-CAT Chinese	Zhong et al., 2013	–	–	NR	NA	+	NR
P-CAT Spanish	Martínez et al., 2016	NR	+	+	NA	–	–
PDC English original	White et al., 2008	+	NR	NR	+	+	NA
PDC American	Sullivan et al., 2012	+	NR	NR	NA	NR	NR
PDC Korean	Choi & Lee, 2014	–	+	NR	NA	+	+
ICS-Nurse Finnish original	Suhonen et al., 2010	+	+	+	+	+	NA
ICS-nurse Swedish	Berg et al., 2012	+	NR	NR	NA	+	+

Note: *NR* not reported, *NA* not applicable

### Categorization according to Tritter’s conceptual framework

In Table 4, we have categorized the scales according to Tritter’s [16] conceptual framework for patient and public involvement. The main types of involvement in the majority of the scales are individual, indirect, and reactive involvement. When categorizing the items, several of them did not fit into any of the categories. Some of them did not reflect patient centeredness or involvement at all; thus, they could not be categorized, and some of them represented individual (e.g., staff attitudes), organizational (e.g., available resources), or environmental (e.g. work environment, climate) preconditions for patient involvement or patient centered practices. The preconditions are items reflecting contextual factors or conditions that either facilitate or are necessary for patient involvement. Two additional rows were added to Table 4 for items that did not fit Tritter’s [16] framework.

**Table 4:** Categorization according to Tritter’s (2009) Framework

Scale (no items)	Type of involvement^a^							
	Individual	Collective	Direct	Indirect	Reactive	Proactive	Not categorized	Preconditions
PCQ – S (14)								14
PCCC (17)	9	0	4	5	5	4	3	5
PCHCOA (31)	9	1	3	7	7	3		21
P-CAT (13)	5	0	0	5	4	1		8
ICS-N (34)	31	0	11	20	27	4		3
PDC (50)	16	2	13	5	9	9	6	26
SEPCQ (27)	9	0	2	7	9	0		18
PC-PAL (62)	10	4	8	6	9	5	14	34
ICI (46)	6	2	2	6	4	4	10	28
PCC (27)	19	0	5	14	12	7	5	3
GCES (28)	3	2	1	4	5	0	6	17

^a^items are categorized according to the following dimensions: individual vs. collective, direct vs. indirect, and reactive vs. proactive

The items in *PCQ* [22] assess the workplace climate rather than involvement, focusing more on staff members than on the patients (e.g., “a place where I feel welcome”). Although some items, such as “a place where it is easy for the patients to talk to the staff,” focus on the patients, they do not include elements of involvement. PCQ is the only instrument in which all items represent preconditions for involvement. All of the included instruments in the review contained items reflecting different forms of preconditions.

The *PCCC* [39] contains items measuring individual involvement, both direct and indirect and reactive and proactive involvement. An example of individual direct and proactive involvement is, “engage patients or designated surrogates in active partnerships that promote health, safety and well-being, and self-care management.” The scale measures several types of preconditions for involvement, for example, “value seeing health-care situations through patients’ eyes” (staff attitude as precondition for patient-centeredness).

Most items in *PCHCOA* [25] represent individual preconditions for involvement, such as staff members’ attitudes and competencies, among others (e.g., “I am supported to develop skills I need to work with older people”). Several items also measure individual indirect and reactive involvement (e.g., “My/our care plans are structured around service user’s goal”) but only three measure direct and proactive involvement (e.g., “The needs and preferences of service users should be central in health services”).

Most *P-CAT* items [24] represent environmental and organizational preconditions for involvement (e.g., “the organization prevents me from providing person-centered care”). An example of individual indirect and proactive involvement is, “we are free to alter work routines based on residents’ preferences.” This scale does not have items measuring collective involvement.

Individual reactive involvement constitutes the main type of involvement in *ICS-N* [21]. Both direct and indirect involvement are represented, but with a predominance of the latter. Items measuring direct involvement are mainly reflected in the “Clin-B-Nurse” dimension, concerning nurses’ perceptions of individuality in care provided. An example of individual indirect and reactive involvement is, “I talk with patients about the feelings they have about their illness/health condition.” No items measure collective involvement, and only a few represent individual preconditions for involvement (e.g., “I give instructions to patients using language that is easy to understand”).

Many of the items in *PDC* [31] represent different forms of preconditions for involvement, including all items in the “knowing the person” dimension (e.g., “know their fears and worries”). This is the only scale with a strong focus on direct involvement compared to indirect involvement, with equal representation of items measuring reactive and proactive involvement. The “autonomy” dimension constitutes most of the items measuring direct and proactive involvement (e.g., “help develop and update care plans, service plans/task lists”).

Two thirds of the items in the *SEPCQ-27* [32] represent individual preconditions for involvement (e.g., “make the patient feel that I have time to listen”). No items assess collective or proactive involvement, and only two items measure direct involvement. Besides the preconditions, most items measure individual indirect and reactive involvement (e.g., “reach agreement with the patient about the treatment plan to be implemented”).

The vast majority of items in the *PC-PAL–staff version* [26] represent different forms for preconditions, most of them belonging to the “workplace practices” dimension (e.g., I’ve received training that helps me assist residents according to their personal preferences and goals). The remaining items reflect mainly individual indirect involvement, with reactive and proactive involvement being equally represented. An example of collective direct and proactive involvement is “residents can suggest, organize, or lead activities and events”.

Most items in *ICI* [30] refer to individual preconditions for involvement (e.g., “I read the social histories of resident care plans”). The remaining items reflect mostly individual indirect involvement with an even split between reactive and proactive involvement. The item, “feel that you are able to allow the residents that you look after to make decisions for themselves,” is an example of direct proactive involvement.

Besides the three items related to preconditions for involvement, all items in the *PCC* [23] measure individual involvement, with the majority being indirect and reactive (e.g., “assess patients health values and goals”), although proactive involvement is also measured to some degree. An example of direct proactive involvement is, “provide the chosen treatment option or self-management strategy”.

Most items in the *GCES* [35] refer to preconditions for involvement (e.g., “lack of specialized services for older adults”). Many of the remaining items were difficult to categorize, as they did not represent any form of involvement or preconditions for involvement (e.g., “differences of opinion among staff (between disciplines) regarding common geriatric problems”). Most of the items that were categorized assessed individual reactive involvement (e.g., “older adults receive the information they need to make decisions about their care”) while none measured proactive involvement.

None of the instruments included items assessing explicitly the use of patients’ experiences for quality improvement. Five of the instruments included some items that might represent possibilities for patients to affect quality practices. However, in all cases where such items were included, they represented indirect involvement in that staff members were required to make suggestions based on their experiences with patient care or input from patients. The following items represents possibilities for patients to influence quality improvement practices:

- Tell my supervisors about the need to change a procedure or practice that is no longer working for resident care (ICI)
- Play a part in the making of facility procedures and practices (ICI)
- Policies and practices for the assisted living community are decided without residents’ input (PC-PAL)^1^
- Engage patients or designated surrogates in active partnerships that promote health, safety and well-being, and self-care management (PCCC)
- Input from staff is sought in determining policies and guidelines about geriatric care (GCES)
- Help develop and update care plans, service plans/task lists (PDC)

## Discussion

Eleven instruments administered to health professionals in different contexts such as hospitals, rehabilitation centers, long-term aged care facilities, inpatients wards, acute care, assisted living, and home care settings, were identified in this review. The analysis of the instruments’ content revealed different types of involvement across the included instruments and items. When categorized according to Tritter’s [16] framework, individual-, indirect-, and reactive involvement were the main types of involvement represented in most scales. Tritter’s [16] framework was not used as a conceptual framework for scale development in any of the articles included in this review. This might explain the lack of items measuring direct and proactive involvement and involvement at a system level, and that several of the items do not fit Tritter’s [16] framework. Patient centeredness scales are not designed to solely measure patient involvement, but we argue that they need to include items that represent patient involvement in order to reflect the interconnectedness between the two constructs. The findings are nevertheless in accordance with Tritter’s [16] model, in that indirect involvement represents the vast majority of involvement activity in healthcare settings.

When analyzing the scales, it became apparent that most scales contained dimensions or items that did not measure actual patient involvement or patient centeredness but rather preconditions for involvement, such as climate and organizational facilitators or barriers. Furthermore, few studies included items explicitly measuring the use of patient experiences for quality improvement. In scales where items reflected possibilities for involvement in such practices, they represented indirect involvement that required the staff members to make suggestions regarding the system development based on their experiences with patient care. This finding might suggest that the recent call for more active involvement of patients in quality and safety improvement [40] needs more attention, especially regarding development of measurement scales reflecting this type of involvement [17]. There is a trend to use measures of patient involvement as part of continuous quality improvement [17, 41], and several studies highlight the benefits of patient involvement in quality and safety in healthcare settings [42–44]. Our results are in line with a recent systematic review concluding that there is a need for valid and reliable strategies for measuring patient involvement as part of continuous quality improvement [17].

Patient involvement in quality improvement goes beyond assessing patients’ experiences of healthcare service and give them possibilities to participate and make decisions concerning their own care [3]. However, in line with our findings, the focus of most studies has primarily been on asking patients what was good or bad with the care they received [45]. Our review supports the notion that relevant tools for healthcare professionals and measurements for using patient experiences in quality improvement are lacking [46]. This is partly due to the lack of consensus regarding how patients can be involved in quality improvement. If we do not know how to involve the patients, it is difficult to know how to measure it. Despite the lack of items explicitly measuring patient involvement in relation to quality improvement practices, patient involvement in their own care and staff listening to patients’ voices (as measured in several of the included instruments in this review) might indirectly affect quality improvement. That is, if staff and management use patients’ experiences to guide quality and patient safety practices. Furthermore, although the different forms of preconditions identified in this review did not fit in to Tritter’s [16] framework, they are still important for making patient-centered practices and patient involvement possible. Davies and Cleary [47] provided several examples of how organizational factors (e.g., lack of supporting values, lack of a quality improvement infrastructure) hinder the use of patient survey data for quality improvement purposes. Such factors also hinder patient centered practices and possibilities for patients to be involved in quality improvement. Therefore, it is important to include them in instruments concerning patient centeredness. Climate and other workplace factors are important for whether and how patients are involved, as well as of the degree to which staff and management are engaged, motivated, and focused on involving patients in healthcare quality improvement.

### Strengths and limitations

The quality of this systematic review was strengthened through close cooperation with a specialized librarian in the search for relevant literature; close cooperation among authors regarding the screening of title, abstract, and full-text as well as the content analysis of the scales; and the critical appraisal of the methodological quality of the scales.

The review has several possible limitations that need to be addressed. We acknowledge that we could have found more studies if we had done a title-, abstract-, and full-text search in addition to using mesh-terms, if we had searched in other databases, and if we did not limit the search to studies published in the 2005–2017 period. However, since our search captured all studies reported in previous literature reviews, in addition to revealing new relevant studies, we argue that the search was relevant and precise. Furthermore, the selected databases are the largest and most relevant for our research field and aim. If studies reporting on person-centered measurement scales were published before 2005, later studies that have used and validated the same scales would probably appear in our search.

Other quality criteria, such as test-retest reliability, convergent validity, and discriminant validity could have been used for a more thorough quality assessment of the included studies. However, the criteria used to assess measurement properties were adapted from the review by Wilberforce et al. [13], as they were believed to cover relevant and key measurement properties. Several of the included scales did not make explicit which definition of patient centeredness they used, and few used a conceptual framework to guide scale development. This is a challenge when assessing whether the scales really measure what they are intended to measure. Articulating a set of theoretical concepts and their interrelations is necessary in order to investigate the construct validity of a measure [19]. Future studies focusing on the development of patient-centeredness or involvement scales should present a clear definition and a theoretical framework guiding the scale development.

The majority of the included studies were of low to moderate methodological quality mainly due to lack of reporting on key measurement properties but also due to low quality of the properties reported. Only Suhonen et al.’s [21] study fulfilled all quality criteria. While this might undermine the validity and applicability of several of the measurement scales included in this review, it highlights a gap in the research literature, calling for further development of high quality person-centered measurement scales.

Using Tritter’s framework has narrowed our focus and we may have overlooked relevant aspects of the content of the scales. The large number of items representing preconditions in most of the instrument indicate that Tritter’s framework may not be well aligned to the scales reviewed. Staff reported instruments tend to include a large number of items looking at organizational approach or readiness (i.e., ‘preconditions’). Thus, basing the analysis on other frameworks such as those proposed by Shaller [48] or Sheard [49] could have be useful for categorizing preconditions of patient centeredness or involvement by type. However, the focus of the content analysis in the current review was on patient involvement, and we have argued why it is reasonable that patient centeredness scales should explicitly include this aspect in the measurement.

### What does this study add?

To our knowledge, this is the first systematic review conducting a directed content analysis of patient-centeredness scales in terms of whether and how patient involvement is included in the scales. Our study adds new knowledge concerning the availability of questionnaire-based scales on patient-centeredness from the staffs’ perspective, how they address the role of patient involvement in quality and patient safety practices, and the categorization of items according to Tritter’s [16] conceptual framework of patient and public involvement (PPC) in health services.

## Conclusions

Individual-, indirect-, and reactive involvement constituted the main involvement types in existing scales on patient centeredness from the staff perspective. Only a limited number of instruments on patient centeredness focus on direct and proactive involvement of patients in quality improvement. Thus, future studies should develop instruments that make the use of patient experiences and patient involvement in quality improvement explicit. Such instruments would be useful for further research on the role of patient involvement in quality improvement in healthcare, and they could also be used as important tools in quality improvement interventions. This review also suggests that a conceptual framework for patient involvement in healthcare, including the role of individual and environmental preconditions for patient involvement, should be further explored and developed.

## Additional file

Additional file 1: *Title of data:* The systematic search and outcomes (medline: May 16., all others: May18. 2017 (2005–2017, English language). *Description of data:* Overview of the systematic literature search strategy in the databases Medline, CINAHL, Embase, and Scopus. The first electronic search was undertaken in April 2017 and updated in May 2017. In each database, the searches were structured around three main concepts: psychometrics, patient-centeredness and involvement, and quality improvement. A combination of keywords, mesh-terms, and subject headings was used in all searches. (DOCX 18 kb)

## Abbreviations

**GCES**: Geriatric Care Environment Scale
**ICI**: Individualized Care Inventory
**ICS-Nurse**: Individualized care Scale
**P-CAT**: Person-centred Care Assessment Tool
**PCC**: Patient-centered care
**PCCC**: Patient-centred care competency
**PCHCOA**: Person-Centered Health Care for Older Adults Survey
**PC-PAL**: Person-Centered Practices in Assisted Living
**PCQ-S**: Person-Centred Climate Questionnaire – Staff version
**PDC**: Person-directed care
**PPI**: Patient and Public Involvement
**PRISMA**: Preferred Reporting Items for Systematic reviews and Meta-Analyses
**SEPCQ-27**: Self-Efficacy in Patient-Centeredness Questionnaire

## Footnotes

1 Negatively worded so that lower scores represent better practices
