# Supplementary material for: How is patient involvement measured in patient centeredness scales for health professionals? A systematic review of their measurement properties and content
Source: BMC Health Serv Res. 2019 Jan 8;19:12. doi: 10.1186/s12913-018-3798-y (PMC6323701; doi:10.1186/s12913-018-3798-y)
Supplement: Supplementary file 1 — Title of data: The systematic search and outcomes (medline: May 16., all others: May18. 2017 (2005–2017, English language). Description of data: Overview of the systematic literature search strategy in the databases Medline, CINAHL, Embase, and Scopus. The first electronic search was undertaken in April 2017 and updated in May 2017. In each database, the searches were structured around three main concepts: psychometrics, patient-centeredness and involvement, and quality improvement. A combination of keywords, mesh-terms, and subject headings was used in all searches. (DOCX 18 kb) [file 12913_2018_3798_MOESM1_ESM.docx]

**Supplemental file 1.** The systematic search and outcomes (medline: May 16., all others: May 18. 2017 (2005-2017, English language))

| ***Database*** | ***Terms*** | ***Hits may. 2017*** | ***Left when removal of duplicates*** | ***Abstract for review (after title exclusion)*** | ***Full text screening*** | ***Included in manuscript*** |
| --- | --- | --- | --- | --- | --- | --- |
| **Medline** | SI. psychometrics (SH*) or reproducibility of results (SH) or validation studies (SH) | 260481 |  |  |  |  |
|  | S2. Patient-centered care (SH) or patient-centred care or person-centred care or person-centered care or individuali*ed care or patient oriented care or client centered care or patient participation (SH) or patient involvement or service user involvement or patient orientation (SH) | 23326 |  |  |  |  |
|  | S3. Quality improvement (SH) or patient safety (SH) or quality of health care (SH) | 2935453 |  |  |  |  |
|  | S1 and S2 | 672 |  |  |  |  |
|  | **S1 and S2 and S3** | **646** | 558 | 71 | 21 | **10** |
| **CINAHL** | SI. Psychometrics (SH) or validation studies (SH) or instrument validation (SH) or reliability and validity (SH) or research instruments (SH) or instrument construction (SH) | 362891 |  |  |  |  |
|  | S2. Patient centered care (SH) or patient-centred care or person-centred care or person-centered care or individuali*ed care or patient orientation (SH) or patient oriented care or client-centred care or consumer participation (SH) or patient involvement or client participation or service user involvement | 21342 |  |  |  |  |
|  | S3. Quality improvement (SH) or quality of health care (SH) or quality of care research (MH) or patient safety (SH) or quality assessment (SH) | 93201 |  |  |  |  |
|  | S1 and S2 | 3780 |  |  |  |  |
|  | **S1 and S2 and S3** | **509** | 500 | 30 | 11 | **3** |
| **Embase** | SI. Psychometry (SH) or internal validity (SH) or reliability (SH) | 245513 |  |  |  |  |
|  | S2. Patient-centred care or patient-centered care or person-centred care or person-centered care or individualized care or individualised care or patient oriented care or client centered care or patient participation (SH) or patient involvement | 22100 |  |  |  |  |
|  | S3. Total quality management (SH) or health care quality (SH) or quality of care or patient safety (SH) |  |  |  |  |  |
|  | S1 and S2 | 796 |  |  |  |  |
|  | **S1 and S2 and S3** | **634** | 502 | 30 | 9 | **4** |
| **SCOPUS** | SI. Psychometry or validation studies or reliability and validity or measurement scale | 106087 |  |  |  |  |
|  | S2. Patient-centred care or patient-centered care or person-centred or individualized care or individualised care or patient orientation or patient participation or patient involvement or client centered care | 32590 |  |  |  |  |
|  | S3. Quality improvement or quality of health care or quality of care or quality improvement or health care quality or patient safety | 212566 |  |  |  |  |
|  | S1 and S2 | 711 |  |  |  |  |
|  | **S1 and S2 and S3** | **135** | **68** | 11 | 5 | **3** |
|  | | | | | | |
| **TOTAL** |  | **1924** | 1628 | 142 | 46 | **20** |

*SH = Subject heading (Medline and CINAHL: MeSH terms, Embase: emtree – a list of subject headings unique to Embase). Duplicates removed: 296
